# Exosomes: The Role in Tumor Tolerance and the Potential Strategy for Tumor Therapy

**DOI:** 10.3390/pharmaceutics15020462

**Published:** 2023-01-30

**Authors:** Yun Lv, Xiaobo Du, Wenqiang Tang, Qian Yang, Feng Gao

**Affiliations:** 1 Departmant of Oncology, NHC Key Laboratory of Nuclear Technology Medical Transformation (Mianyang Central Hospital), Mianyang Central Hospital, School of Medicine, University of Electronic Science and Technology, Mianyang 621000, China; 2 Department of Oncology, Affiliated Hospital of North Sichuan Medical College, Nanchong 637503, China; 3 Center of Scientific Research, Chengdu Medical College, No. 783, Xindu Avenue, Xindu District, Chengdu 610500, China

**Keywords:** exosomes, drug resistance, radiotherapy resistance, engineered exosomes

## Abstract

Drug and radiotherapy resistance is the primary cause of treatment failure and poor prognosis in patients with tumors. Exosomes are extracellular vesicles loaded with substances such as nucleic acids, lipids, and proteins that transmit information between cells. Studies have found that exosomes are involved in tumor therapy resistance through drug efflux, promotion of drug resistance phenotypes, delivery of drug-resistance-related molecules, and regulation of anti-tumor immune responses. Based on their low immunogenicity and high biocompatibility, exosomes have been shown to reduce tumor therapy resistance by loading nucleic acids, proteins, and drugs inside xosomes or expressing tumor-specific antigens, target peptides, and monoclonal antibodies on their phospholipid bimolecular membranes. Consequently, future research on genetically engineered exosomes is expected to eliminate resistance to tumor treatment, improving the overall prognosis of patients with tumors.

## 1. Introduction

The global tumor burden has increased over the past few decades. In China, the national relative rate of cancer-related deaths increased from 10.1% in the 1970s to 24.2% in 2015 [1]. The tumors with the highest incidence are related to lung, stomach, liver, colorectum, and bladder cancers in men and breast, lung, colorectal, thyroid, cervical, and stomach cancers in women. More than 600,000 people in the U.S. will die from cancer by 2022 [2]. In Europe, there were more than 3.9 million new cancer cases, of which 53% were male, and 47% were female, and the total number of cancer deaths was estimated at 1.93 million in 2018 alone [3]. Therefore, cancer is currently the main health problem that needs to be managed. 

The natural progression of tumors includes gene mutations that can lead to the malignant proliferation of tumor cells, destruction and remodeling of the surrounding microenvironment by relative molecules released from tumor cell clusters, entry into the circulatory system through the wall of blood and lymphatic vessels, colonization and growth in distant organs, and, ultimately, death of the patient. Based on an in-depth understanding of tumor occurrence and development, current anti-tumor therapies include traditional surgery, chemotherapy, radiotherapy, emerging immunotherapy, targeted therapy, chimeric antigen receptor (CAR) T-cell therapy, and ionizing radiation. However, in addition to the early patients who were cured by surgery, most patients with malignant tumors have primary therapy resistance or would develop therapy resistance at a certain stage of tumor treatment, resulting in a poor prognosis. Different solutions can be used for different types of therapy resistance. Solutions to single-agent chemotherapy resistance include the use of drugs with different mechanisms of action, different dose intensities, shorter chemotherapy intervals, or higher doses supplemented with growth factor support. One of the solutions to targeted therapy resistance is to identify new gene mutation sites and use the corresponding targeted drugs [4]. Unfortunately, eventual resistance to drugs and radiotherapy remains a typical occurrence. Therefore, we focus on the specific molecular functions of exosomes involved in tumor therapy resistance and the regulation of exocrine function, which have potential applications in disease treatment.

## 2. Biological Functions of Exosomes for Cellular Communication

**Figure 1:**
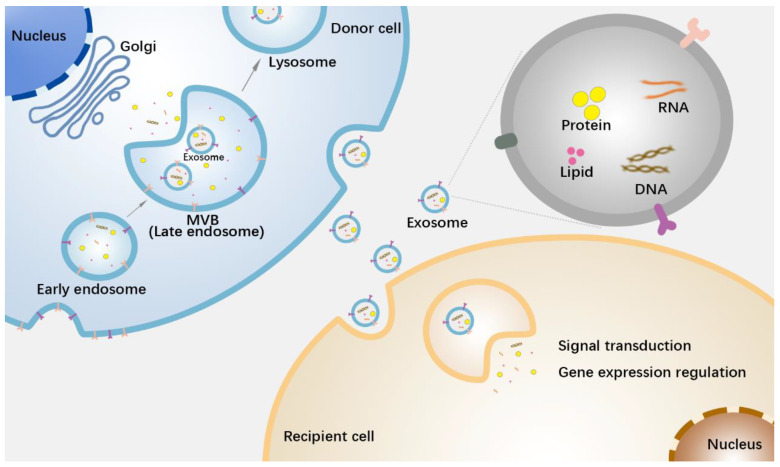
Exosomes transmit information between cells. Exosomes originate from early endosomes and are loaded with substances such as nucleic acids, proteins, and lipids in the process of forming intracellular multivesicular bodies (MVBs). MVBs then transport the exosomes out of the cells by fusing with the cell membrane. After entering the recipient cells, exosomes release the components involved in signal transduction or gene expression regulation [6,7,20].

Exosomes, extracellular vesicles (EVs) with diameters ranging from approximately 40–160 nm, are released by nearly all cells. Pan et al. first identified exosomes in sheep reticulocytes in 1983, used to track the transferrin receptor during maturation [5]. Reportedly, a variety of cells can secrete exosomes, such as nerve cells, immune cells, epithelial cells, mesenchymal cells, and tumor cells [6]. The formation of exosomes is a delicate and complex biological process. First, cells form early endosomes through endocytosis, which contain extracellular small molecules and cell membrane surface proteins. These early endosomes then form late endosomes through double invagination of the plasma membrane, eventually forming intracellular multivesicular bodies (MVBs), in which intraluminal vesicles (ILVs) of different diameters, namely exosomes, are generated. A variety of specific molecules in the cytoplasm are loaded into exosomes through the endosomal sorting complex required for transport (ESCRT)-dependent or -independent mechanism, and ultimately, the exosomes are secreted outside the cell through the fusion of these MVBs with the cytoplasmic membrane [7,8,9]. Exosomes enter receptor cells through endocytosis, direct fusion, or combination with surface receptors. In recent decades, exosomes have attracted much attention because they act as cell-signaling mediators by transferring proteins, RNA, DNA, lipids, and other substances between cells. Interestingly, the composition of the exosomes was not variable (Figure 1). There is growing evidence that exosomes loaded with different contents are involved in various complex physiological and pathological processes, including tissue development [10], immune response [11,12,13], reproductive health [14], autophagy [15], cardiovascular disease [16], and cancer progression [17,18,19]. In terms of physiological and pathological mechanisms, exosomes derived from tumor cells, tumor-associated immune cells (e.g., tumor-associated macrophages), and tumor-associated stromal cells (e.g., tumor-associated fibroblasts) can promote angiogenesis, remodel the tumor microenvironment, regulate anti-tumor immune responses, and induce resistance to tumor growth, invasion, and metastasis [20]. For example, exosomes with the epidermal growth factor receptor EGFRvIII, released from glioma cells, can be taken up by EGFRvIII-deficient cancer cells, activating translational signaling pathways and enhancing growth ability [21]. Tumor cells successfully induce angiogenesis by delivering the exosome Tspan8-CD49d complex into endothelial cells [22]. In addition to providing exosomes, tumor cells can also “educate” adjacent stromal cells to remodel the tumor microenvironment. Breast cancer exosomes can increase the level of the unshielded protein RN7SL1 in stromal fibroblasts, which is secreted by stromal cells with exosomes, driving inflammatory responses in the microenvironment and promoting tumor cell growth, metastasis, and therapy resistance [23]. 

## 3. Isolation and Purification of Exosomes

Exosomes can be identified in almost all biological fluids, including blood, urine, bronchoalveolar lavage fluid, lacrimal fluid, seminal fluid, and ascites [24,25]. However, one of the challenges in the clinical application of exosomes is that their isolation and storage are not standardized. A variety of methods have been developed to isolate exosomes; however, most of the available isolation techniques are unable to obtain large quantities of high-purity exosomes while maintaining vesicle integrity. Differential ultracentrifugation uses different centrifugal forces and times to sort materials based on the density and size of exosomes and other components [26,27]. After the removal of dead cells and cell debris from the cell culture supernatant or biological fluid, the exosome particles are suspended in PBS solution and can be used immediately for biological experiments or stored in a refrigerator at 4 °C for near-term use. This technique has been successfully used to isolate exosomes from cell culture supernatant, serum, saliva, urine, breast milk, and amniotic fluid [27,28]. Although this method is simple and easy to operate, the process takes a long time, can partially damage exosomes, and is vulnerable to the influence of centrifugation time and biological material type [29]. Based on these shortcomings, isopycnic density-gradient centrifugation, one-step sucrose cushion-buffered centrifugation [29,30], and cushioned-density gradient ultracentrifugation have all been developed. Since the marker proteins expressed on the exosome membrane surface include CD9, ALIX, an-nexin, and Rab5, immunoaffinity capture technologies based on magnetic beads or affinity columns have been developed. The main principle is to use magnetic beads or affinity columns coated with antibodies to capture exosomes from exosome suspensions enriched in ultracentrifugation by recognizing specific signaling receptors on the exosome membrane surface. This technique is suitable for isolating specific exosome subgroups for further study of their biological functions; however, the low yield of this method limits their further development and clinical use [31].

Precipitation techniques use polymers such as polyethylene glycol to separate exosomes from liquids [32]. Several precipitation-based commercial products are available for purchase. However, it should be noted that polyethylene glycol is difficult to separate from exosomes and has potential toxicity. The chitosan separation method has also been used to collect exosomes [33]. Positively charged chitosan can attract negatively charged exosomes, thereby separating them from the cell culture medium, urine, blood, and saliva. This technique can safely isolate intact exosomes at a relatively low technical cost. However, the elution of exosomes from chitosan-exosome complexes may be less efficient given the differences in density and pH of different biological fluids. Based on the size difference among exosomes and other components of biological fluids, exosomes can be separated using cellulose membranes with different molecular weight cut-off values, a method called ultrafiltration. This method is simple and less time-consuming, but the clogging of exosomes on the surface of the membrane may lead to a decrease in recovery [34]. Researchers have successfully isolated different exosome subpopulations using the simple, rapid, and gentle asymmetric flow–field flow fractionation, which is expected to help researchers further elucidate the heterogeneity and biological origin pathways of exosomes. However, the small capacity of gentle asymmetric flow–field flow fractionation makes it difficult to use for large-scale preparation of exosomes [35]. Microfluidic techniques can separate exosomes based on exosome-specific proteins or lipids (label-based) or physical properties of exosomes (label-free). The label-based microfluidic method captures exosomes based on the principle of antigen-antibody specific binding, but the dissociation process of the antigen-antibody complex may destroy the exosome structure. In contrast, label-free microfluidic techniques can better ensure the intact structure and biological composition of exosomes. Furthermore, the simple operation steps, high cost-effectiveness, and short time consumption make label-free microfluidic techniques a very promising method for exosome isolation. However, this method may not be able to distinguish exosomes from lipoproteins of similar size and density [36]. The efficient exosome detection method via the ultrafast-isolation system (EXODUS) is an efficient exosome purification method that improves the separation efficiency by incorporating double-coupled harmonic oscillations in the dual membrane filter. The researchers compared EXODUS with other exosome isolation methods, including ultracentrifugation, size-exclusion chromatography, and polyethylene glycol precipitation. The results revealed that EXODUS was able to obtain high-purity exosomes and their subpopulations at a faster rate and in higher yields, which offers the possibility of large-scale preparation of exosomes and future clinical applications [37].

All of the above methods have advantages and disadvantages. It is necessary to continue to develop simple, efficient, and low-cost separation strategies and gradually standardize them to ensure the accuracy of results, further meeting the needs for sustainable medical care.

## 4. Exosomes Participate in Resistance of Tumor Therapy

With the exploration of exosomes, their various functions in tumor therapy resistance are gradually being clarified.

### 4.1. Exosomes Reduce Intracellular Drug Concentration through Drug Efflux

The mechanisms of tumor drug resistance involve tumor burden, physical barriers, tumor heterogeneity, immune dysregulation, and selective therapeutic pressure. Exosomes are involved in tumor therapy resistance processes through various complex mechanisms.

Some anti-tumor drugs need to enter the cytoplasm or nucleus for subsequent anti-tumor effects; however, tumor cells can sort intracellular drugs into exosomes and then secrete them outside the cells. Lehuédé et al. found that breast cancer cells co-cultured with breast adipose cells developed a resistance to doxorubicin (DOX). Further studies revealed that DOX accumulated in the exosomes of these breast cancer cells and was subsequently secreted extracellularly in conjunction with an increase in the transport-associated major vault protein [38]. Similarly, studies have shown that cisplatin accumulates in the lysosomes of tumor cells, and cisplatin-resistant ovarian cancer cells secrete more exosomes carrying lysosome-associated proteins 1 and 2 and cisplatin into the extracellular environment [39]. B-cell lymphoma cells were observed to extrude the anthracycline, DOX, and the anthracenedione, pixantrone, from cells by secreting exosomes, which may be related to the involvement of the vital molecule ATP transporter A3 in exosome biogenesis [40]. In addition, tumor cells block the delivery of anti-tumor drugs by constructing an acidic microenvironment, which further leads to drug resistance. In an acidic environment, the uptake of cisplatin by human melanoma cells is significantly reduced. These tumor cells sort cisplatin into exosomes and then discharge drugs from the cells, resulting in drug resistance [41]. 

### 4.2. Exosomes Promote Tumor Cells to Develop Drug-Resistant Phenotype

**Figure 2:**
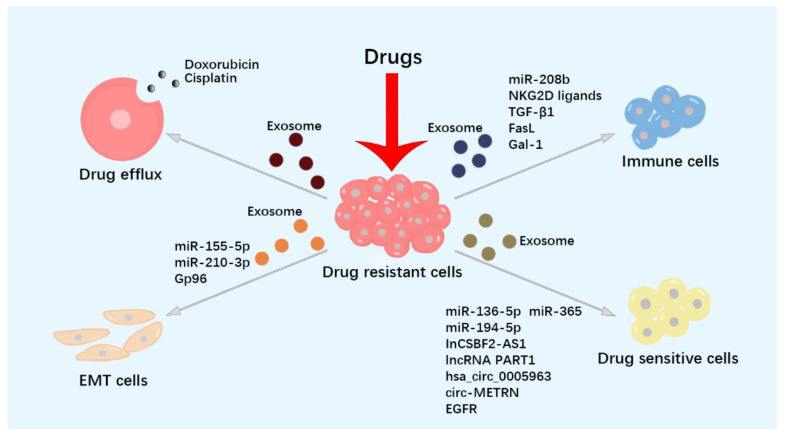
Exosomes participate in tumor drug resistance. Exosomes participate in tumor drug resistance by directly mediating drug efflux, regulating anti-tumor immune responses, inducing epithelial–mesenchymal transition (EMT) phenotype, and delivering drug resistance [39,40,41,42,43,47,48,53,54,55,60,61,66,77,79,80,81,91,95].

EMT is a flexible change in cellular phenotypes, during which epithelial cells lose adherence junctions, acquire mesenchymal properties, and develop invasion and drug resistance. MicroRNAs (miRNAs) can influence gene expression levels through recognition sites in the 3′-untranslated region of a specific mRNA. Some exosomes involved in resistance to tumor therapy are listed in Table 1. Elevated miR-155-5p expression was found in paclitaxel-resistant gastric cancer, with an epithelial-to-mesenchymal transition (EMT) phenotype (Figure 2, Table 1). Sensitive cells exhibited the EMT phenotype after the uptake of paclitaxel-resistant cell-derived exosomal miR-155-5p. Further research revealed that miR-155-5p could target GATA-binding protein 3 (GATA3) in drug-sensitive tumor cells [42]. Exosomes released from oncogenically transformed mesenchymal human bronchial epithelial cells (HBECs) transfer chemoresistance to epithelial HBECs and increase the expression of the EMT transcription factor ZEB1, resulting in gemcitabine and cisplatin resistance of the recipient cells [43]. Clinically, increased circulating exosomal miR-92a-3p levels in patients with colorectal cancer (CRC) are associated with chemotherapy resistance. Exosomal miR-92a-3p, isolated from cancer-associated fibroblasts (CAFs), inhibited FBXW7 and MOAP1 expression, increased cell stemness, and induced EMT and 5-FU resistance [44]. Exosomal miR-155 collected from breast cancer stem cells (CSCs) and chemoresistant cells could be transferred to recipient-sensitive cells and effectively induce EMT change, DOX, and paclitaxel drug resistance [45]. Exosomal gp96 from paclitaxel-resistant breast cancer cells increases paclitaxel resistance in paclitaxel-sensitive breast cancer cells by degrading p53. Hypoxia promotes EMT and paclitaxel resistance in tumor cells [46]. Furthermore, exosomal miR-210-3p may play a role in osimertinib resistance by inducing the EMT process in the tumor microenvironment of EGFR-mutant non-small cell lung cancer (NSCLC) [47].

### 4.3. Exosomes Deliver Drug-Resistant-Associated Molecules

Studies have found that exosomes can also transmit drug resistance to cancer cells. Exosomes derived from cisplatin (DDP)-resistant triple-negative breast cancer (TNBC) cells alter the sensitivity of other tumor cells by delivering miR-423-5p [48]. Exosomes with miR-21 released from cisplatin-resistant oral squamous cell carcinoma (OSCC) could induce cisplatin resistance and promote tumor growth, as indicated in the subcutaneous xenograft mouse model [49]. Exosomal miR-4443 from cisplatin-resistant NSCLC is transferred to sensitive cells and confers drug resistance by regulating FSP1 [50]. A significant decrease in miR-567 was found in trastuzumab-resistant HER-2 positive breast cancer patients over that found in responding patients. Overexpressed exosomal miR-567 can be taken in by recipient-resistant cells, suppress autophagy, and reverse trastuzumab resistance by targeting ATG5 both in vitro and in vivo [51]. As a molecularly targeted tyrosine kinase inhibitor specific to EGFR, osimertinib has been widely used for EGFR-mutant NSCLC. However, most advanced NSCLC patients treated with osimertinib develop drug resistance and tumor progression within 1 year. A previous study showed that osimertinib promotes the secretion of exosomes from EGFR-non-mutation-resistant lung cancer cells. These exosomes then transferred wild-type EGFR protein to sensitive cells, which induced osimertinib resistance by activating the PI3K/AKT signaling pathways [52]. Moreover, a report indicated that exosomal miR-136-5p from anlotinib-resistant advanced NSCLC cells induced anlotinib resistance in sensitive NSCLC cells by targeting PPP2R2A [53]. In addition to the tumor cells, stromal cells in the tumor microenvironment can also deliver drug-resistant miRNAs. Tumor-associated macrophages (TAM) from the pancreatic ductal adenocarcinoma (PDAC) microenvironment secrete exosomes carrying miR-365. miR-365 upregulates triphospho-nucleotides in PDAC cells and significantly decreases the sensitivity of tumor cells to gemcitabine [54]. Furthermore, miR-100, miR-222, and miR-433 can be loaded into exosomes and involved in chemotherapy resistance [55,56].

Long noncoding RNA (lncRNAs) that biochemically resemble mRNAs are defined as RNA genes with base pairs larger than 200 yet do not have protein-coding potential. lncRNAs can compete with endogenous RNA (ceRNAs) to regulate miRNA expression levels and form “lncRNA–miRNA–mRNA” axes [57]. Exosomal lncARSR released from renal cell carcinoma (RCC) cells promotes sunitinib resistance by binding to miR-34 and miR-449. Sunitinib-resistant RCC cells treated with an AXL/c-MET inhibitor showed restored sunitinib sensitivity [58]. The alkylated drug temozolomide (TMZ) is a standard chemotherapeutic drug for malignant glioma treatment; however, drug resistance greatly increases the difficulty of clinical treatment. Exosomal lnCSBF2-AS1 from TMZ-resistant glioblastoma (GBM) cells upregulated X-ray repair cross complementing 4 expression levels by targeting miR-151a-3p, enhancing drug resistance in GBM cells [59]. Furthermore, high levels of serum exosomal lncRNA PART1 in patients with esophageal squamous cell carcinoma (ESCC) are clinically associated with adverse reactions to gefitinib treatment. The lncRNA PART1 from gefitinib-resistant cells promotes Bcl-2 expression in parental ESCC cells in vitro by binding to miR-129 and promoting gefitinib resistance [60]. lncRNA-SNHG14 incorporated into exosomes from trastuzumab-resistant HER-2+ breast cancer cells can disseminate resistance to sensitive cells by targeting the apoptosis regulator Bcl-2 (Bcl-2) signaling pathway [61]. lncRNA XIST, lncRNA UCA1, and lncRNA AX747207 [62,63,64] can also be selectively loaded into exosomes of tumor cells and participate in tumor drug resistance.

Circular RNAs (circRNAs) are produced by post-splicing the precursor mRNA of gene exons in eukaryotes. Drug-resistant tumor cells can improve the energy metabolism of sensitive cells by delivering circRNAs. Oxaliplatin-resistant CRC cells transferred the circular RNA hsa_circ_0005963 (ciRS-122), which is a sponge for pyruvate kinase (PKM2)-targeting miR-122, to sensitive cells through exosomes. CiRS-122 in recipient cells upregulates the expression of PKM, which is a key molecule in catalyzing glycolysis, and gradually transforms cells into drug-resistant cells [65]. CircRNA nuclear factor I X in exosomes, released from TMZ chemoresistant glioma cells, was found to repress cell apoptosis under TMZ exposure and enhance cell migration and invasion by sponging miR-132 in recipient-sensitive cells [66]. Exosomal circ_0072083 expression is increased in TMZ-resistant glioma patients. Circ_0072083 silencing can reduce NANOG expression by blocking demethylation and restraining TMZ resistance in tumor cells [67]. Additionally, exosomal circ 0000338, circ CPA4, and circ PVT1 are also involved in the process of tumor chemotherapy resistance [68,69,70].

Hormonal therapy is an endocrine therapy for tumors, including postmenopausal estrogen receptor-positive breast cancer. Estrogen-dependent MCF-7 breast cancer cells showed partial antiestrogen drug resistance after treatment with exosomes derived from tamoxifen- and/or biguanide-metformin-resistant cells. The transmission of this resistance is partly related to the activation of Akt, NF-κB, and SNAIL1 transcription factors [71].

N 6-methyladenosine RNA demethylase FTO was found to be enriched in circulating exosomes collected from gefitinib-resistant advanced NSCLC patients, as compared to gefitinib-sensitive patients. FTO reduction in exosomes from gefitinib-resistant cells alleviated the acquired resistance of the gefitinib-sensitive cell line PC-9 cells by the FTO/YTHDF2/ABCC10 axis in vitro and in vivo [72]. Multidrug resistance (MDR) leads to poor response to clinical chemotherapy in some tumor patients. A previous study indicated that docetaxel resistance in prostate cancer could be partly due to the transfer of one of the key genes related to drug resistance, MDR-1, via exosomes [73]. Moreover, mesenchymal stem cell exosomes upregulate MDR-associated proteins, such as MDR and LRP, by activating the Raf/MEK/ERK kinase cascade in cancer cells and play a promoting role in drug resistance to 5-fluorouracil [74].

### 4.4. Exosomes Regulate Anti-Tumor Immune Response

Immunosuppression is one process by which continuous tumor progression proceeds. One study indicated that murine mammary tumor cell-derived exosomes inhibited the release of perforin from NK cells, blocking their cytotoxic response and promoting tumor growth [75]. NKG2D is an activating receptor of immune cells, and its abnormal loss leads to tumor immune evasion. Cancer cell exosomes expressing NKG2D ligands and TGF-β1 downregulate NKG2D expression and weaken the ability of CD8(+) T and NK cells to recognize and kill tumor cells [76]. Murine mammary tumor cell exosomes block the differentiation of bone marrow (BM) CD11b (+) myeloid precursor cells into dendritic cells (DC), resulting in the accumulation of myeloid precursors in mouse spleen. Moreover, this study suggested that IL-6 and phosphorylated Stat3 play important roles in blocking immune cell differentiation [77]. A study found that circulating exosomal miR-208b is a potential biomarker for oxaliplatin resistance prediction in patients with CRC. Exosomal miR-208b derived from colon cancer cells promoted regulatory T cell (Treg) expansion by targeting programmed cell death factor 4 (PDCD4) once taken in by recipient T cells, leading to tumor growth and oxaliplatin resistance in vivo [78]. FasL-positive (FasL+) exosomes have been detected in the serum of patients with oral squamous cell carcinoma. These microvesicles promote T lymphocyte apoptosis by activating mitochondrial apoptotic pathways [79]. Exosomes with galectin-1 (Gal-1) from head and neck cancer-derived cells have been shown to induce an immune suppressor phenotype in human CD8+ T cells, resulting in the immune escape of tumor cells [80]. 

At present, monoclonal antibodies have become one of the primary means of targeted anti-cancer therapy, owing to immune-mediated lytic mechanisms in tumor cells. Monoclonal antibodies as a targeted anti-cancer therapy have benefited most patients with different tumor types, and the anti-CD20 chimeric antibody rituximab has been widely used. Rituximab exerts anti-tumor effects by inducing cytolysis after CD20 ligation. However, the prognosis for patients with primary drug resistance remains poor. A study found that exosomes with the CD20 antigen released from aggressive B-cell lymphoma cells could bind rituximab and protect lymphoma cells from humoral immunotherapy. Exosomes carrying the CD20 receptor on the lipid membrane derived from B-cell lymphoma can bind to the anti-CD20 antibody rituximab, thereby mediating its extracellular depletion [81]. Trastuzumab is a humanized antibody used as an adjunctive therapy for breast cancer patients with HER-2 overexpression. Unfortunately, exosomes derived from breast cancer cells can bind trastuzumab, isolating tumor cells from the drug and reducing drug availability [82]. Drug resistance associated with lncRNA actin filament-associated protein 1 antisense RNA 1 (AFAP1-AS1) may lead to a shorter survival time in a fraction of HER-2-positive breast cancer patients. Mechanistically, AFAP1-AS1 loaded in exosomes from trastuzumab-resistant cells induces trastuzumab resistance by promoting ERBB2 translation.

### 4.5. Exosomes Transfer Radiation Resistance

Radiotherapy is a method of treating malignant tumors using radiation, including α-, β-, and γ-rays, produced by various X-ray therapy machines or accelerators. Radiation can be absorbed by tumor cells and directly or indirectly damages the DNA of cells, resulting in cell death. However, both radiation-sensitive and radiation-resistant cells exist in the tumor microenvironment, and the existence of these radiation-resistant cells is one of the reasons for tumor recurrence and progression. Exosomes are important signal transduction carriers in the tumor microenvironment and play a notable role in tumor radiation resistance.

Radiotherapy causes DNA damage in cancer cells, resulting in changes in the quantity and composition of the exosomes. A study showed that radiotherapy induced a p53-dependent increase in exosomes with the B7-H3 protein in human prostate cancer cells, which was identified as a diagnostic marker for prostate cancer [83]. Similarly, GBM cells and normal astrocytes secreted more exosomes after precise X-ray exposure. These exosomes are absorbed by recipient cells and enhance their migration ability by activating neurotrophic tyrosine kinase receptor type 1 (TrkA) [84]. Jelonek et al. compared the components of exosomes derived from human squamous head and neck carcinoma FaDu cells exposed to ionizing radiotherapy. They found that exosomes from FaDu cells exposed to ionizing radiation carried different proteins involved in transcription, translation, and cell signaling [85].

As information carriers, exosomes transmit radiation resistance between tumor cells. Researchers have found that radiation-resistant cells can enhance the enrichment of h3k4me2 to express more noncoding RNA NORAD, which can inhibit miR-199a-5p expression and reduce the content of miR-199a-5p in exosomes. However, NORAD knockdown increased the expression level of miR-199a-5p in exosomes, inhibiting the ATR/Chk1 signaling pathway and restoring the radiosensitivity of radiation-resistant cells. Moreover, NORAD knockdown increased the efficacy of radiotherapy and anti-PD-1 treatment in mice by inhibiting PD-L1 ubiquitination [86]. Radiation can enhance the release and uptake of exosomes by tumor cells, and these exosomes not only facilitate unirradiated cell proliferation but also promote the survival of irradiated cells. These large numbers of exosomes from irradiated cells transmit radiation resistance among cells, possibly through increased DNA double-strand break repair [87]. Increased circulating exosomal circRNA was found to be associated with disease recurrence in patients with nasopharyngeal carcinoma, and overexpressed circMYC could reduce the radiosensitivity of tumor cells [88]. Hypoxia is a critical factor in radiation resistance. Exosomes can transmit radiation resistance between hypoxic and aerobic cells. MiR-340-5p is highly expressed in exosomes released from hypoxic esophageal squamous cell carcinoma (OSCC) cells. Exosomal miR-340-5p is taken up by aerobic cells and then targeted to KLF10/UVRAG, resulting in radiation resistance [89]. Clinical studies have shown that circMETRN can be detected in the serum exosomes of patients with GBM at the early stage of radiotherapy. Further studies have shown that low-dose radiotherapy can cause tumor cells to produce exosomes carrying high levels of circMETRN and circMETRN-induced radiation resistance through the miR-4709-3p/GRB14/PDGFRα pathway [90]. A report demonstrated that exosomes released from latent membrane protein 1 (LMP1)-positive recipient nasopharyngeal carcinoma (NPC) cells induce radiation resistance in recipient NPC cells, partly via activation of P38 MAPK signaling in the recipient cells. Notably, these exosomes promote cell migration and invasion while inducing radiation resistance in recipient cells [91]. In addition to tumor cells, exosomes secreted by tumor-associated stromal cells are also involved in tumor radiation resistance. CAFs are malignant cells assimilated by tumor cells. Exosomes derived from CAFs have been found to promote the clonogenicity and radiation resistance of CSCs in CRC by activating the TGF-β signaling pathway [92]. Furthermore, M2-like TAMs have been detected in clinical endometrial carcinoma (EC) tissue samples. Hsa_circ_0001610 in exosomes released from TAMs significantly downregulated the radiosensitivity of EC cells by upregulating cyclin B1 expression in a competitive manner with miR-139-5p [93]. A report indicated that exosomal miR-194-5p released from irradiated tumor cells potentiated the survival of residual tumor-repopulating cells (TRCs) after radiotherapy by inducing G1/S arrest in pancreatic cancer [94].

The radiation-induced bystander effect and radiation-induced abscopal effect refer to a biological response in which irradiated cells influence adjacent and distant unirradiated cells through intercellular signal transduction. A study found that HaCaT skin keratinocytes exposed to α-particles and X-rays secrete exosomes containing more miR-27a. Exosomal miR-27a was taken in by recipient unirradiated WS1 skin fibroblasts and inhibited migration of WS1 cells by targeting MMP2 [95]. Autophagy occurs in recipient cells receiving exosomal miR-7-5p from irradiated human bronchial epithelial BEP2D cells via the EGFR/Akt/mTOR signaling pathway [96]. Human prostate cancer PC3 cells secrete exosomes containing L-plastin after exposure to ionizing radiation. Exosomal L-plastin is constitutively Ser5-phosphorylated in malignant cancer and normal cells and induces the reduction of mitogenic/clonogenic activity [97]. Compared with the control group, exosome-like vesicles in the serum of four Gy-irradiated mice carried amplified mitochondrial DNA (ND1, ND5), which could induce DNA damage in fibroblasts [98]. Similarly, exosomes released by irradiated cells activated ATM and ATR, which impaired DNA replication in recipient FaDu cells [99]. 

**Table 1:** Exosome components involved in cancer therapy resistance.

	Exosome Components	Resistance	Effects	References
Gastric cancer	miR-155-5p	Paclitaxel	Induce EMT	[43]
Breast cancer	miR-155	Doxorubicin and paclitaxel	Induce EMT	[46]
Non-small cell lung cancer	miR-210-3p	Osimertinib	Induce EMT	[48]
Non-small cell lung cancer	miR-136-5p	Anlotinib	Targeting PPP2R2A	[54]
Pancreatic ductal adenocarcinoma	miR-365	Gemcitabine	Up-regulate the triphospho-nucleotide	[55]
Colon cancer	miR-208b	Oxaliplatin	Promote regulatory T cells expansion	[79]
Pancreatic cancer	miR-194-5p	Radiotherapy	Induce G1/S arrest	[95]
Glioblastoma	InCSBF2-AS1	Temozolomide	Up-regulate X-ray repair cross complementing 4	[60]
Esophageal squamous cell carcinoma	lncRNA PART1	Gefitinib	Up-regulate Bcl-2	[61]
Colorectal cancer	hsa_circ_0005963	Oxaliplatin	Up-regulate PKM	[66]
Endometrial carcinoma	hsa_circ_0001610	Radiotherapy	Up-regulate cyclin B1	[94]
Glioma	circ-nuclear factor I X	Temozolomide	Repress cell apoptosis	[67]
Glioblastoma	circ-METRN	Radiotherapy	Activate miR-4709-3p/GRB14/PDGFRα pathway	[91]
Aggressive B-cell lymphoma	CD20 antigen	Rituximab	Bound rituximab	[80]
Lung cancer	Wild type EGFR protein	Osimertinib	Activate PI3K/AKT and MAPK signaling pathways	[53]

## 5. Modification of Exosomes for Tumor Therapy

**Figure 3:**
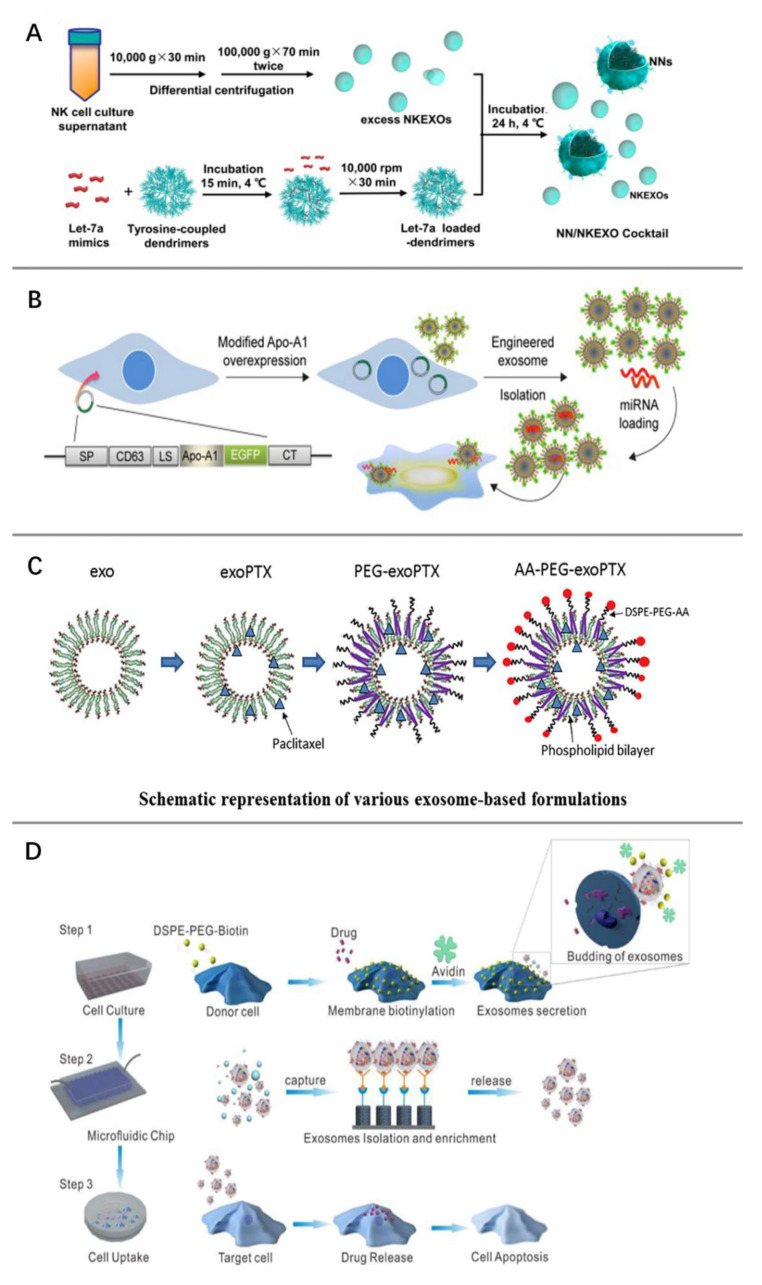
Modified exosomes for tumor therapy. (**A**) Co-culture of natural killer cell-derived exosomes (NKEXOs) with therapeutic-loaded let-7a nanoparticles (NNs) produced a cocktail that inhibits neuroblastoma cell growth. Reprinted with permission from Ref. [103]. 2019, CC BY. (**B**) Engineered exosomes that can specifically bind to hepatoma cells were obtained by loading miR-26a into exosomes from 293T cells overexpressing scavenger receptor class B type 1 (Apo-A1) through electroporation.Reprinted with peimisson from Ref. [107].2018, CC BY. (**C**) Targeting of exosomes to sigma receptor-expressing lung cancer was enhanced by expressing aminoethylanisamide-polyethylene glycol (AA-PEG) on the phospholipid bilayer plasma membrane of paclitaxel-loaded macrophage-derived exosomes (exoPTX). Reprinted with permission from Ref. [108]. 2018, Elsevier. (**D**) Microfluidic chips were used to efficiently isolate and collect biotin and avidin-labeled drug-encapsulated exosomes, which were taken up by recipient cells and induced apoptosis. Reprinted with permission from Ref. [111]. 2017, American Chemical Society.

With the unveiling of the physiological and pathological mechanisms of exosomes, novel tumor therapeutic strategies using exosomes themselves or as delivery agents are being actively explored. Traditional drug delivery systems are represented by liposomes, which are non-toxic spherical vesicles composed of the phospholipid bilayer wrapped around a water core and have been successfully used for the delivery of many drugs [100]. However, the clearance rate of liposomes by the reticuloendothelial system is high, and there is a risk of liposomes triggering hypersensitivity reactions. Exosomes, as naturally secreted vesicles of cells, are emerging as a very promising tool for anti-tumor drug delivery based on their low immunogenicity and excellent biocompatibility. There are several strategies available to load therapeutic drugs, proteins, or nucleic acids into exosomes. Electroporation is an effective method to passively load exogenous substances into exosomes. The use of specific electric fields can temporarily increase the permeability of exosome membranes, at which time substances are loaded into the exosomes, and the exosome membranes are then restored to integrity [101]. Co-culture of cisplatin with tumor cells can produce vesicles containing cisplatin that can be preferentially taken up by tumor-repopulating cells. These vesicles reverse chemoresistance in tumor-repopulating cells by interfering with drug efflux [102]. Likewise, engineered exosomes loaded with therapeutic miRNAs produced by co-culture delivered miRNAs to neuroblastoma cells in vivo, thereby inhibiting tumor growth [103] (Figure 3A). 

In addition, several other strategies for exosome loading have been compared: freeze-thaw cycles, saponin permeabilization, extrusion, or sonication. It was found that exosomes generated by extrusion, sonication, or saponin permeabilization had the highest loading efficiency [104]. Exosomes successfully loaded with specific substances are able to increase the sensitivity of tumor cells to drugs. Adipose tissue-derived mesenchymal stem cells cultured with miR-122 secrete miR-122-encapsulated exosomes, which increased the sensitivity of hepatoma cells to sorafenib by delivering miR-122 to hepatoma cells [105]. Tumor-cell-derived engineered CRISPR/Cas9 exosomes enhance the chemosensitivity of ovarian cancer cells to cisplatin [106]. 

In addition to molecular loading, improving exosome targeting is also important. Genetic engineering and chemical modifications can improve the tumor cell targeting specificity of exosomes. Genetic engineering enables the expression of this specific protein on the exosome surface via cloning the gene sequence of the specific protein to the gene sequence of the exosome transmembrane protein. Cloning of the Apo-A1 gene into the downstream sequence of the CD63 in donor cells produced exosomes that expressed Apo-A1 on the surface of bilayer lipid membranes, and these exosomes were specifically recognized by scavenger receptor class B1 positive hepatocellular carcinoma cells, thereby enhancing the tumor targeting of the exosomes. These exosomes were then loaded with therapeutic miR-26a by electroporation. The modified exosomes were confirmed to inhibit the growth and invasion of cancer cells [107] (Figure 3B). The sigma receptor is a membrane-bound protein highly expressed in lung cancer cells, but its role in cancer remains unclear. Exosomes modified with 1, 2-Distearoyl-sn-glycero-3-phosphoethanolamine-N-polyethylene glycol-aminoethylanisamide (DSPE-PEG-AA) and loaded with paclitaxel can target tumor cells expressing the sigma receptor and inhibit tumor cell lung metastasis. DSPE-PEG-AA and paclitaxel were loaded on macrophage-derived exosomes by a special sonication method, and this modification protected PTX from mononuclear phagocyte system clearance and prolonged the circulation time [108] (Figure 3C). 

Similarly, the gene sequences encoding GE11 or EGF were cloned into pDisplay vectors to produce exosomes expressing GE11 or EGF membrane proteins. Engineered exosomes target tumor cells through specific binding of GE11 or EGF to EGFR on the surface of cancer cells [109]. Chemical modifications are equally capable of loading ligands onto the surface of exosomes. Neuropilin-1-targeted peptide (RGERPPR, RGE) can be conjugated to exosome membranes through click chemistry, which enables curcumin-carrying exosomes to target glioma cells [110]. Donor cells are labeled with biotin and anti-biotin proteins using a chemical editing strategy and loaded with the drug in the cytoplasm. Microfluidic microarray technology was then used to isolate exosomes secreted by such cells that carry anti-cancer drugs and express biotin and anti-biotin proteins. These exosomes have been proven to be highly effective in targeting tumor cells and enhancing the effect of anti-cancer drugs [111] (Figure 3D). In addition, artificially synthesized extracellular nanovesicles have recently been developed and are expected to become a new generation of drug delivery systems [112,113,114]. 

As most tumors are immunosuppressed, activation of the immune system is an effective anti-tumor strategy. As early as 1996, exosomes from B lymphocytes were found to carry major histocompatibility complex (MHC) class II molecules and participate in antigen presentation in vivo [115]. Dendritic cell-derived exosomes express T-cell costimulatory molecules, MHC class I, and class II molecules. These exosomes can activate T cell-mediated immune responses in vivo and suppress tumor growth [116]. Subsequently, molecules such as liposome-associated membrane protein 1 (LAMP1), CD81, and rab7 were also found to be enriched in exosomes [117,118]. In addition to antigen presentation function, exosomes can stimulate the migration of natural killer cells [119], promote the proliferation of T cells [120], and enhance the anticancer immune response of helper T cells [121]. Modified exosomes have also been used to activate anti-tumor immune responses. Exosomes with CpG on the surface of the lipid membrane can activate Toll-like receptor 3, eliminating chemotherapy resistance and immunosuppression and enhancing the anti-tumor immune response to advanced ovarian cancer in vivo. Prostate-specific antigen (PSA) and prostatic acid phosphatase (PAP) were loaded onto exosomal membranes by fusing them to the lactadherin protein, which increases the antigenicity of PSA, enhances the immune response to PAP in mice with prostate cancer, and improves the anti-tumor effect. In another study, exosomes expressing anti-human HER2 antibodies were genetically engineered to attack HER2-positive breast cancer cells via activating cytotoxic T cells [122]. It should be noted that exosomes can also promote tumor immune escape by suppressing the immune response. Some exosomes could impair NK cell cytotoxicity, inhibit T cell function, inhibit DC activity, promote macrophage M2 polarization, and increase the proliferation of myeloid-derived suppressor cells [123]. 

The feasibility of exosomes to inhibit cancer progression and stimulate anti-tumor immune responses in vivo makes exosome-based cancer vaccines a new cancer treatment strategy. Exosome-based vaccines have been proven to inhibit tumor growth, stimulate Th1 immune responses, and suppress tumor metastasis [124,125]. Due to immunosuppression in the tumor microenvironment, exosome-based vaccines alone produce limited antitumor immune responses. Therefore, exosome-based vaccines in combination with antitumor drugs are an effective strategy to improve anti-tumor responses. Tumor exosome-loaded dendritic cell vaccination combined with all-transretinoic acid, sunitinib, and gemcitabine inhibited myeloid-derived suppressor cells, increased the number of activated T cells, and prolonged the survival time [126]. Increasing the immunogenicity of exosomes can improve the efficacy of cancer vaccines. For example, compared to untreated exosomes, heat-treated exosomes are enriched with heat shock proteins, which stimulate TH1 immune responses in vivo and exert anti-tumor effects [127]. In addition, for breast cancer patients who are resistant to the anti-HER2 monoclonal antibody trastuzumab, a novel HER2-specific exosome-T vaccine was developed using exosomes released from HER2-specific dendritic cells to target CD4+ T cells. The new vaccine bypassed HER2 tolerance and primed the immune system of mice against HER2-positive breast cancer [128]. Other modification strategies for cancer vaccines have been well-reviewed [123]. Several clinical trials have also been conducted for exosome vaccines [129,130,131,132]. However, currently, the FDA has approved TheraCys^®^ for early bladder cancer, PROVENGE^®^ for castration-resistant prostate cancer, and IMLYGIC^®^ for metastatic melanoma.

## 6. Conclusions and Perspectives

In conclusion, resistance to therapy remains the greatest challenge in cancer treatment. The mechanisms of resistance to tumor therapy are complex and constantly change with tumor development and treatment. As a type of messenger, exosomes have an extensive influence on tumor cell therapy resistance, including direct drug efflux, induction of EMT, promotion of immune escape, and delivery of therapy-resistant-associated molecules. Fully understanding the signal transduction between exosomes in the tumor microenvironment and cells may help reduce therapy resistance, improve the therapeutic effect on malignant tumors, and improve the prognosis of tumor patients. With an in-depth understanding of exosomes, researchers have begun to pay attention to their potential in translational medicine, such as in disease diagnosis [133,134,135] and therapeutic application [136,137,138]. Detection of proteins and nucleic acids loaded in exosomes or exosome surface proteins in saliva, blood, and body fluids can assist in disease diagnosis, tumor staging, fetal sex determination, and patient prognosis prediction [139]. However, there is no consensus on the use of exosomes as a standard for the diagnosis and prognosis of diseases. Given the ability of exosomes to carry peptide-MHC complexes and activate immune cells, several engineered exosomes have emerged as tumor vaccines. These exosomes were artificially modified to enhance their immunogenicity and induce potent anti-tumor effects in vivo [140]. In some research institutions, exosomes are exploited and engineered as drugs for the treatment of diseases and tumors [141,142]. However, the drug-loading capacity and targeting specificity of exosomes need to be further optimized. Among the current exosome loading strategies, extrusion, sonication, or saponin permeabilization showed excellent loading efficiency. However, whether exosomes derived from different types of cells have the same cargo delivery ability is not yet clear. 

Finding ways to improve the production and purity of exosomes is also an important cornerstone for the future clinical transformation of exosomes. At present, there is no single method of exosome isolation that can be applied to all studies. Therefore, researchers may consider choosing one of the most appropriate or combining two methods to obtain exosomes, depending on the purpose of the study. For the future clinical application of exosomes, it is necessary to consider the ability to handle large volumes of clinical samples and the reliability of the results. Based on this, ultrafiltration, label-free microfluidic techniques, and EXODUS have great potential to become the technical support for the clinical application of exosomes. In the future, it will be necessary to comprehensively explore the biological functions, pharmacokinetics, toxicology, and other aspects of exosomes and promote relevant clinical trials to better apply exosomes in clinical diagnosis and treatment.

## Data Availability

Not applicable.
